# Expression, function, and regulation of the testis-enriched heat shock *HSPA2* gene in rodents and humans

**DOI:** 10.1007/s12192-014-0548-x

**Published:** 2014-10-25

**Authors:** Dorota Scieglinska, Zdzislaw Krawczyk

**Affiliations:** Maria Skłodowska-Curie Memorial Cancer Center and Institute of Oncology, Gliwice Branch, Wybrzeże Armii Krajowej 15, 44-101 Gliwice, Poland

**Keywords:** Heat shock genes, HSPA2, Spermatogenic cells, Somatic cells, Cancer, Cytoprotection, Regulation of expression

## Abstract

The *HSPA2* gene is a poorly characterized member of the *HSPA* (*HSP70*) family. *HSPA2* was originally described as testis-specific and expressed at the highest level in pachytene spermatocytes of rodents, the expression of which is not induced by heat shock. *HSPA2* is crucial for male fertility. However, recent advances have shown that *HSPA2* is expressed in various tumors and in certain types of somatic tissues. In this review, we summarize the current knowledge on the *HSPA2* expression pattern, including information on transcriptional, translational, posttranslational, and epigenetic mechanisms which regulate *HSPA2* expression. We also present and discuss the current views concerning the functions of the HSPA2 protein in spermatogenetic, somatic, and cancer cells. The knowledge of the properties of HSPA2, although limited, shows this protein as a unique member of the HSPA family. However, understanding whether this protein could become a relevant cancer biomarker or a therapeutically applicable target requires extensive further studies.

## Introduction

More than 50 years ago, studies of Ferrucio Ritossa (Ritossa 1962) paved the way for exciting research which resulted in the discovery of a cellular reaction known as the heat shock response. As we know now, this is a highly universal response of almost all kinds of cells and all species exposed to factors/conditions that could affect the normal structure of cellular proteins. The major feature of the heat shock response is the rapid induction or considerable stimulation of the expression of genes encoding heat shock proteins (HSPs), the function of which is to minimize the harmful effects of environmental or endogenous molecular stressors (Gidalevitz et al. 2011). As a result of long-term studies, multiple HSPs and HSP-related proteins have been identified which are classified (mostly on the basis of similar molecular weights and amino acid sequence similarity) into several families. Guidelines for the nomenclature of the human HSPs have been recommended by Kampinga et al. (2009), and in this review, we will follow them.

The general function of HSPs, regarded as molecular chaperones, is their involvement in the maintenance of intracellular homeostasis, primarily by controlling the process of protein folding. Many HSPs act as molecular partners for other highly specialized proteins such as signal transducers and transcription factors. HSPs also exhibit anti-apoptotic properties and, as well, can modulate various immune responses. All these properties make HSPs of prime interest in various fields of medicine as potential clinically useful markers or targets for therapeutic intervention (Kakkar et al. 2014; Ciocca et al. 2013).

The largest group of heat shock proteins is the HSPA (HSP70) family (Table 1), which in humans contains at least 13 members, only some of which are highly heat/stress inducible (Kampinga et al. 2009). Within the HSPA family, there are two genes, namely *HSPA2* and *HSPA1L*, which were originally identified as highly specific for spermatogenesis. The aim of this review is to summarize the current knowledge on *HSPA2* gene expression and regulation as well as to discuss possible functions of the gene in spermatogenic, normal somatic, and cancer cells.

**Table 1:** Genes belonging to the HSPA family in human genome

Name	Other names	Locus	Entrez gene ID	Protein size (molecular mass kDa)	Protein homology (%)^a^
*HSPA1A*	HSP70-1; HSP72; HSPA1	6p21.33	3303	641 (70,0)	83.5
*HSPA1B*	HSP70-2^b^	6p21.32	3304	641 (70,0)	83.5
*HSPA1L*	hum70t; hum70t; Hsp-hom	6p21.33	3305	641 (70,4)	82
*HSPA2*	Hsp70-2^b^, Hsp70.2	14q23.3	3306	639 (70,0)	100
*HSPA5*	BIP; GRP78; MIF2	9q33.3	3309	654 (71,0)	60.9
*HSPA6*	Heat shock 70 kD protein 6 (HSP70B′)	1q23.3	3310	643 (71,0)	78.3
*HSPA7*		1q23.3	3311	ND	ND
*HSPA8*	HSC70; HSC71; HSP71; HSP73	11q24.1	3312	646 (70,9)	86.3
*HSPA9*	GRP75; HSPA9B; MOT; MOT2; PBP74	5q31.2	3313	679 (73,7)	46
*HSPA12A*	FLJ13874; KIAA0417	10q25.3	259217	675 (141,0)	13.1
*HSPA12B*	RP23-32L15.1; 2700081N06Rik	20p13	116835	686 (75,7)	14.5
*HSPA13*	Stch	21q11.2	6782	471 (51.9)	23.8
*HSPA14*	HSP70-4; HSP70L1; MGC131990	10p13	51182	509 (54,8)	28.3

*ND* no data

^a^Amino acid homology to the protein encoded by HSPA2

^b^Please note that in the literature, the same name was sometimes used for two different genes, e.g., *HSPA1B* and *HSPA2*

## Cloning and structure of the *HSPA2* gene

The first clue suggesting the existence of a testis-specific hsp70-related gene was the detection in total RNA isolated from rat testis of a highly abundant 2.7-kb transcript which hybridized with DNA probes derived from *Drosophila* or human heat-inducible *hsp70* genes and with mouse genomic DNA sequences (then unidentified) cloned into the pM1.8 plasmid (kindly given to us by Dr. Rick Morimoto) (Krawczyk et al. 1987b). The expression pattern of the 2.7-kb transcript during the seminiferous epithelium cycle and postnatal testis development, and the disappearance of this transcript from testis of rats exposed to factors which impaired spermatogenesis with concomitant degeneration of seminiferous epithelium, indicated the corresponding gene to be highly and selectively activated in pachytene spermatocytes (Krawczyk et al. 1987a; Krawczyk et al. 1988; Krawczyk and Szymik 1989). The gene was isolated from a rat genomic library, cloned, and named *Hst70* (Wisniewski et al. 1990). The current name is *HSPA2* (Entrez Gene ID: 60460).

In a parallel study, Zakeri and Wolgemuth (1987) found in the testis of adult mice a similar 2.7-kb transcript, which hybridized with a DNA probe corresponding to the murine heat-inducible *hsp70*/*hsp68* gene. This transcript was abundantly expressed in postmeiotic early spermatids but was barely detectable in prophase spermatocytes. Such an expression pattern suggested that this particular transcript was coded rather by a spermatid-specific gene. This gene was characterized later and named *Hsc70t* (current name; *HSPA1L*, Entrez Gene ID: 15482) by Matsumoto and Fujimoto (1990)*.*

The mouse counterpart of the rat *hst70*/*HSPA2* gene was identified by Zakeri et al. (1988) by screening a mouse DNA genomic library with the above-mentioned pM1.8 plasmid as a probe*.* Cloning and sequencing of the murine ortholog of the rat *hst70*/*HSPA2* gene (then named *Hsp70-2*; Entrez Gene ID: 15512) confirmed that its 5′ end fragment was present in the pM1.8 genomic DNA clone (Zakeri et al. 1988).

The structure of the transcription unit of the rat and mouse *HSPA2* gene is similar. They have an intron within the 5′ untranslated region and two main transcription start sites (T2 and T1) located at around 116 base pairs (bp) and around 351-bp upstream of the ATG codon (numbers related to the position in the rat gene) respectively (Widłak et al. 1994, 1995; Dix et al. 1996b; Scieglinska et al. 2001). Accordingly, two variants of the *HSPA2* transcripts are synthesized in mouse and rat testis, both having a similar size (2.7 kb) but a different structure of their 5′ end. The population which originates from the distant T1 transcription start site undergoes splicing. The second, non-spliced variant of messenger RNA (mRNA) originates from the T2 start site (approx. 116-bp upstream of ATG) placed within the intron (Scieglinska et al. 2001).

The human *HSPA2* gene (Gene Entrez ID: 3306), which has been cloned from a human placenta genomic library (Bonnycastle et al. 1994), encodes a protein with 98.2 and 98.4 % amino acids (aa) sequence similarity to its mouse and rat counterparts, respectively. The most conspicuous difference between the human and rodent HSPA2 protein is the insertion of 7 aa near the carboxyl end (aa 623–629) of human HSPA2. Transcription of the human *HSPA2* gene initiates at a single transcription start site, which corresponds to the T2 site of the rodent ortholog gene (placed 109-bp upstream of the ATG codon) (Piglowski et al. 2007). The human gene, localized at 14q24.3 (Bonnycastle et al. 1994), has no intron(s) and its expression gives rise to only one population of mRNA molecules (Piglowski et al. 2007).

The human and rodent *HSPA2* genes show the highest amino acid sequence similarity (86.3 %) to the *HSPA8* gene. According to a recent study, in which phylogenetic trees of the human *HSPA* genes have been computed based on the alignment of their protein products, *HSPA2* together with *HSPA8* groups into one of three subgroups of one of the seven major evolutionary-related groups of *HSPA* genes. A second subgroup of this group includes *HSPA1A*, *HSPA1B*, and *HSPA1L* genes while the third one includes *HSPA6* and *HSPA7* genes (Brocchieri et al. 2008). Evolutionary analysis presented by Brocchieri et al. 2008 also suggested that the sequence of *HSPA2*, *HSPA1*, and *HSPA6* genes originated from the *HSPA8* gene by retrotransposition.

## Expression pattern of the *HSPA2* gene in normal somatic and pathological tissues

Although initially the *HSPA2* gene was described as testis-specific, subsequent studies revealed that it could be expressed also in somatic tissues. The first hint which pointed to this came from the study of transgenic (TG) mice, because a transgene composed of a chloramphenicol acetyltransferase (CAT) reporter gene fused to the rat *HSPA2* promoter was expressed not only in the testis, but also in the brain (Widłak et al. 1995). The expression of the *HSPA2* gene in the mouse brain, at the highest level in the hippocampus, was subsequently shown by Northern blotting and in situ hybridization (Murashov and Wolgemuth 1996b). The use of the more sensitive RT-PCR method enabled detection of low levels of *HSPA2* mRNA present in multiple rat and mouse tissues, except liver (Scieglińska et al. 1997; Dix et al. 1996b). Activity of the *HSPA2* promoter was also observed during mouse embryogenesis (Murashov and Wolgemuth 1996a; Rupik et al. 2006).

A study aimed to identify cell types which express the *HSPA2* gene was performed in TG mice bearing the transgene composed of the rat *HSPA2* promoter and an enhanced green fluorescent protein (*EGFP*) reporter. Visualization of EGFP fluorescence in TG mice tissues and detection of endogenous HSPA2 protein (by Western blot and immunohistochemistry) indicated that *HSPA2* expression is restricted to certain types of tissues and specific cells (Vydra et al. 2009; Table 2).

**Table 2:** Summary on the expression of the *HSPA2* gene in selected human and mouse tissues

	Human HSPA2; Gene ID: 3306					Mouse HSPA2 (Hsp70.2); Gene ID: 15512			
	Northern blot^a^	BioGPS^b^	EST^c^	Protein^d^	Cell type^d^	BioGPS^e^	EST^c^	Protein^f^	Cell type^f^
Adrenals	nd	19.6	60	+	Cortex—zona reticularis cells	43.8	0	+	Medulla—chromaffin cells
Bladder	nd	nd	0	−	–	190.5	61	+	Muscularis; urothelium—umbrella cells
Lung	+	230.9	17	+	Bronchial epithelium	57.5	30	+	Bronchial epithelium
Heart	+	34.4	67	−	–	20.7	0	−	–
Brain	++	2189.0	641	+	Glial cells, ependymal cells	145.4	73	+	Cortex—pyramidal cells; cerebellum—Purkinje cells; dentate gyrus—granular and molecular layers; hippocampus—mossy fiber; ependyma; pia mater, choroid plexus, subcommissural organ; neurons in subventricular zone
Colon	++	369.1	76	+	Epithelium—goblet cells; smooth muscle fibers of lamina muscularis mucosae	18.8	nd	nd	nd
Kidney	++	252.4	198	+	Cortex—distal tubules	18.4	0		Parietal layer of Bowman’s capsules
Liver	–	33.6	4	−	–	18.0	0	−	–
Ovary	++	9.9	9.0	nd	nd	89.5	0	+	Oocytes from primodial and primary follicles, *follicle—granulosa cells*, ovarian mesothelium
Placenta	++	229.0	687.6	nd	nd	32.9	nd	nd	nd
Prostate	+	51.2	21	−	–	52.6	0	+	Cuboidal epithelial cells
Skeletal muscle	+++	29.8	27	−	–	25.0	36	nd	nd
Skin	nd	19.2	147	+	Epidermis—basal layer	nd	58	+	Hair bulbs, epidermis
Small intestine	++	185.7	nd	+	Epithelium—goblet cells, paneth cells; smooth muscle fibers of lamina muscularis mucosae	38.6	nd	+	Epithelium—goblet cells; muscularis, enteric ganglia
Spleen	+	nd	92	−	–	13.1	10	+	Peyer’s patches
Testis	+++	2276.0	553	+	Spermatocytes and spermatids	6748.9	344	+	Spermatocytes and spermatids
Thyroid	nd	44.6	0	−	–	nd	0	nd	nd

^a^Results according to Bonnycastle et al. (1994); *nd* no data, *+* weak, *++* moderate, *+++* strong hybridization signal

^b^Data on expression of human HSPA2 extracted from BioGPS database; expression values obtained for 211538_s_at probe set; *nd* no data

^c^Number of ESTs corresponding to the *HSPA2* gene per million transcripts in the given tissue; data on relative EST number extracted from the UNIGENE database (www.ncbi.nlm.nih.gov/unigene); *nd* no data

^d^Results of protein expression in human tissues according to Scieglinska et al. (2011); *nd* no data, *+* positive detection, *−* negative detection

^e^Data on expression of human HSPA2 extracted from the BioGPS database; expression values obtained for 1417101_at probe set; *nd* no data

^f^Results of protein expression in mouse tissues according to Vydra et al. (2009); *nd* no data, *+* positive detection, *−* negative detection

The finding of abundant anti-sense transcript (2.8 kb) in somatic mouse tissues, detected mainly in various brain structures (Murashov and Wolgemuth 1996a, b), suggests a high complexity of the *HSPA2* expression pattern in rodents. However, at present, a functional meaning of this anti-sense RNA is entirely obscure.

The first study on the expression of *HSPA2* mRNA in human tissues showed an abundant *HSPA2* transcript in a majority of somatic tissues with the exception of liver and peripheral blood leukocytes (Bonnycastle et al. 1994; Table 2). Subsequent studies confirmed the expression of *HSPA2* mRNA in numerous immortalized non-tumorigenic cell lines derived from breast, bronchus, and prostate (Rohde et al. 2005; Piglowski et al. 2007; Scieglińska et al. 2008), although not in cell lines derived from human embryonic kidney (Hageman et al. 2011) and normal urothelium (Garg et al. 2010b). The search for HSPA2 protein performed by immunohistochemistry using multitissue microarrays and a highly specific anti-HSPA2 anti-serum revealed that beside testicular cells, HSPA2 is expressed in several tissues in a cell-type-specific manner (Scieglinska et al. 2011; Table 2).

The bioinformatic data available in the BioGPS (www.biogps.org) and UNIGENE (www.ncbi.nlm.nih.gov/unigene) databases support the view that the *HSPA2* gene can be expressed in multiple, but not all, somatic tissues. UNIGENE predicts approximate expression patterns by assessing the relative number of expressed sequence tags (ESTs) per tissue, while BioGPS collects data obtained through analysis of high-density oligonucleotide arrays. According to these databases, the highest level of *HSPA2* transcripts can be found in the testis and brain of both human and mouse. In other tissues, the mRNA is present at substantially lower levels, if any (Table 2). However, the pattern of *HSPA2* gene transcription in human and mouse somatic tissues is not fully overlapping, and the relative abundance of the *HSPA2* mRNA is significantly higher in human than in mouse somatic tissues (Table 2).

The expression of the *HSPA2* gene, both at protein and mRNA levels, was also demonstrated by several groups in cancer cell lines originated from various human malignancies as well as in primary malignant tumors (details in Table 3). Our recent immunohistochemical study with the use of tissue microarrays demonstrated that the HSPA2 protein is widely expressed in human malignancies, although the percentage of HSPA2-positive samples may vary between different tumor histotypes (Scieglinska et al. 2014). For instance, HSPA2 was found in a majority of skin, breast, or lung squamous cell cancer (SCC) cases, but only in a minority of prostate and lung adenocarcinomas (Scieglińska et al. 2008, 2014). Because high HSPA2 expression was associated with negative prognosis in esophageal SCC, non-small cell lung cancer (NSCLC), and liver hepatocellular cancer (HCC) patients (Zhang et al. 2013; Scieglinska et al. 2014; Fu et al. 2014), it seems that this protein could be a clinically relevant cancer biomarker (Table 3). We discuss the possible influence of HSPA2 on cancer phenotype in the later section of this review (“Function of HSPA2 in normal somatic and cancer cells” section).

**Table 3:** Summary on HSPA2 expression in cancer cell lines and human primary metastatic tumors

Organ	Cell lines	HSPA2 expression mRNA/protein		Tumors	HSPA2 (results of IHC)	Clinical significance
Bladder	Cancer: HTB-1^a^, UMUC-3^a^, HTB-9^a^, HTB-2^a^	+/+		Urothelial cancer	Expression in 80 % of tumors (*n* = 116, PS)^a^	Increased expression associated with tumor progression^a^
	Normal urothelial cell line^a^	−/−			Overexpression in 21 % of tumors (*n* = 19, TMA)^i^	
Breast	Cancer: MCF-7^b,c,d^	+/+		Breast cancer	Expression in 63 % of tumors (*n* = 27, TMA)^i^	nd
	Non-tumorigenic: HBL-100^b,c,d^, MCF-10^b,d^	+/+				
Cervix	Cancer: SiHa^e^, CaSki^e^, C-33 A^e^, HeLa^b,c,e^	+/+		Cervical SCC	Expression in 86 % of tumors (*n* = 76, PS)^b^	Increased expression associated with tumor progression^e^
Colon	Cancer: LoVo-36^b^	−/nd		Colon AC	Expression in 47 % of tumors (*n* = 17, TMA)^i^	nd
	Cancer: HCT-116^d^	+/−				
Esophagus	nd	nd		Esophageal SCC	Expression in 75 % of tumors (*n* = 120, PS)^k^	Expression associated with primary tumor, TNM stage, lymph node metastases, and recurrence^k^
						Expression correlated with shorter patients OS^k^
Germ cell	Teratocarcinoma: NTERA2^f^	nd/+		nd	nd	nd
Liver	Cancer: HepG2^d,g^	−/−^d^	+/+^g^	Liver HCC	High expression in 57 % of tumors (*n* = 119, PS)^j^	Expression related to tumor size, differentiation, and stage^j^
	Cancer: HUH7^b^	+/nd			Expression in 17 % of tumors (*n* = 18, TMA)^i^	High expression correlated with shorter patients OS^j^
Lung	Cancer: NCI-H1299^d^, A549^d^, NCI-H358^d^	+/+		Lung NSCLC	Expression in 62 % of tumors (*n* = 85, PS)^i^	Nuclear expression associated with histology and TNM stage^i^
	Non-tumorigenic: BEAC-2B^d^	+/+				High expression correlated with shorter patients OS^i^
Prostate	Cancer: PC3^b,h^	+/+		Prostate AC	Expression in 6 % of tumors (*n* = 16, TMA)^i^	nd
	Non-tumorigenic: PNT1A^b^	+/+				

*IHC* immunohistochemistry, *PS* paraffin-embedded postsurgical tumor samples, *TMA* tissue microarray, *SCC* squamous cell carcinoma, *AC* adenocarcinoma, *HCC* hepatocellular carcinoma, *NSCLC* non-small cell lung carcinoma, *OS* overall survival, *nd* no data, *+* positive detection, *−* negative detection

^a^Results according to Garg et al. 2010b

^b^Results according to Rohde et al. 2005

^c^Results according to Daugaard et al. 2007

^d^Results according to Scieglińska et al. 2008

^e^Results according to Garg et al. 2010a

^f^Results according to Sasaki et al. 2008

^g^Results according to Huang et al. 2009

^h^Results according to Alekseev et al. 2011

^i^Results according to Scieglinska et al. 2014

^j^Results according to Fu et al. 2014

^k^Results according to Zhang et al. 2013

An important issue facing HSPA researchers concerns obtaining antibodies highly specific for a particular member of the HSPA family. The major problem is cross-reactivity of a given anti-HSPA antibody with several HSPA proteins due to very high amino acid sequence similarity between them. This problem pertains even to commercial, commonly used anti-HSPA antibodies (Chow et al. 2010; Scieglinska et al. 2011). Table 4 collects data on antibodies used for HSPA2 detection. So far, rabbit polyclonal anti-sera raised in accordance with a protocol developed by Rosario et al. (1992) against a short peptide derived from the C-terminal part (aa 611–628) of mouse HSPA2 have been the most commonly used (Son et al. 1999; Rohde et al. 2005; Daugaard et al. 2007; Scieglińska et al. 2008, 2011, 2014; Garg et al. 2010a, b). It is worth mentioning that, due to the presence of a glycine-rich 7 aa insertion (GGGGAGA after serine 623) in the C-terminal part of human HSPA2, a corresponding peptide should not be considered as an antigen for generation of anti-HSPA2 antibodies. This insertion makes the C-terminal part of human HSPA2 highly similar to other glycine-rich proteins (Scieglinska et al. 2011).

**Table 4:** Examples of antibodies used for HSPA2 detection

Host/Clonality	Antigen	Purity	Source	Reference
R/P	M, bovine thyroglobulin-conjugated peptide (aa 611–628)^b^	–	Custom-made	Son et al. (1999)^a^; Huszar et al. (2000)^a^; Alekseev et al. (2009)^a^
R/P	M, bovine thyroglobulin-conjugated peptide (aa 611–628)^b^	Affinity purified using protein extract from human testis	Custom-made	Scieglinska et al. (2008, 2011, 2014)
R/P	M, ovalbumin-conjugated peptide (aa 611–628)^b^	–	Custom-made	Rohde et al. (2005); Daugaard et al. (2007)
R/P	M, ovalbumin-conjugated peptide (aa 611–628)^b^	Affinity purified using protein G	Custom-made	Garg et al. (2010a, b)
R/P	H, ovalbumin-conjugated peptide (aa 611–627)^c^	–	Custom-made	Huang et al. (2009)
R/P	H, sperm HSPA2 excised from preparative 2-D gels	–	Custom-made	Naaby-Hansen and Herr (2010)
R/P	H, protein fragment (aa 488–637)^c^	Affinity purified using antigen	Sigma-Aldrich (HPA000798)	Redgrove et al. (2013)
M/P	H, full-length recombinant protein (aa 1–639)^c^	Purified immunoglobulin	Sigma-Aldrich (SAB1405970**)**	Redgrove et al. (2013)
M/Mo	H, full-length recombinant protein (aa 1–639)^c^	Protein G purified	Abcam (ab55290)	Alekseev et al. (2011); Wu et al. (2011)
R/P	H, full-length HSPA2 isolated from spermatozoa	–	Custom-made	Huszar et al. (2000)
M/Mo	H, recombinant protein	–	Santa Cruz Biotech (JJ-3)	Zhang et al. (2013)
G/P	H, peptide mapping within internal region of HSPA2 (aa not specified)	Affinity purified	Santa Cruz Biotech (K-12)	Fu et al. (2014)

*H* human, *M* mouse, *G* goat, *Mo* monoclonal, *P* polyclonal, *R* rabbit

^a^Anti-serum obtained by Rosario et al. 1992

^b^NCBI Reference Sequence NP_001002012.1

^c^NCBI Reference Sequence NP_068814.2

The antigen affinity purification of the rabbit polyclonal HSPA2 anti-serum, raised in accordance with Rosario’s protocol, improved both the sensitivity and specificity of detection. Such purified anti-HSPA2 antibody showed no cross-reactivity with recombinant HSPA1, HSPA6, and HSPA8 (the most similar to HSPA2) proteins (Scieglińska et al. 2008, 2011). To the best of our knowledge, no extensive verification of the specificity of other anti-HSPA2 antibodies (Table 4), including possible cross-reaction with other HSPA members, has been published.

## Regulation of the expression of the *HSPA2* gene

### DNA regulatory elements required for HSPA2 expression in rodent testis

**Fig. 1:**
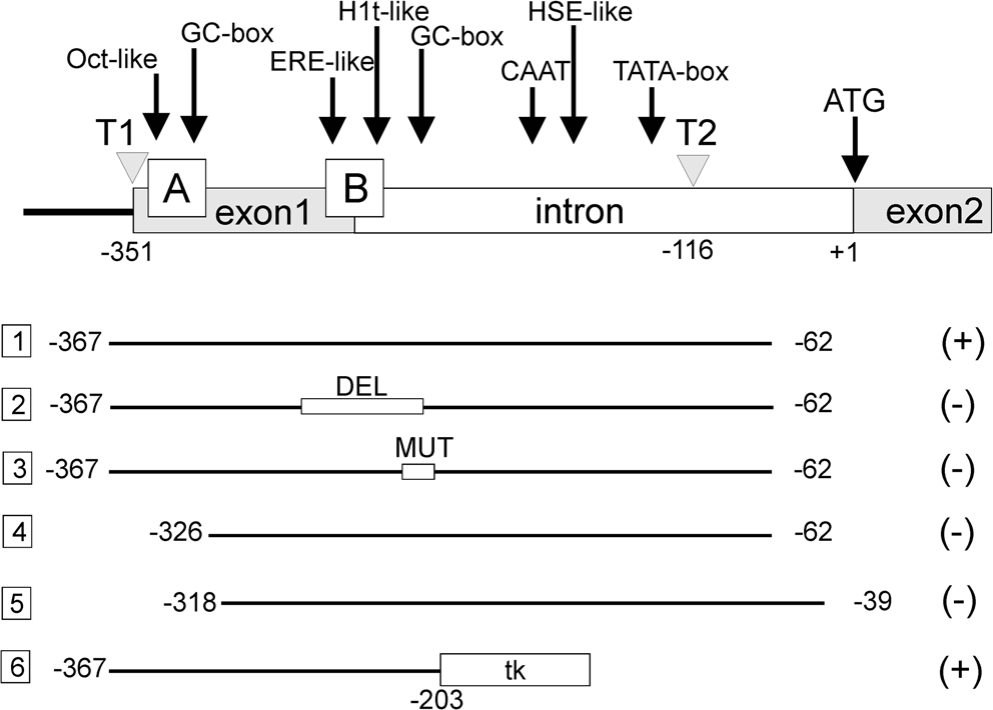
Summary of results concerning functional analysis of the activity of the HSPA2 promoter in transgenic mice testes. The *upper diagram* shows the structure of the HSPA2 transcription unit. The potential sequences implicated in regulation of the HSPA2 gene expression in rodent tissues are indicated. Note that only the regulatory sequences that were subjected to functional studies are shown. T1 (−353) and T2 (−116) transcription start sites. The *bottom diagram* shows the promoter fragments that were fused to a reporter gene and analyzed in transgenic mice testes. Transgene expression (*positive*); No transgene expression (*negative*). Activities of constructs 1 and 4 were reported in Scieglinska et al. (2001), those of constructs 2 and 3 were described in Widlak et al. (2007a), construct 5 was evaluated in Dix et al. (1996b), and finally, construct 6 was assessed in Scieglińska et al. (2004). *Numbers* are coordinates of DNA fragment position in relation to A (+1) in the ATG translation start codon. *DEL* deletion, *MUT* point mutations, *tk* thymidine kinase promoter

The *HSPA2* gene, initially classified as unique, testis-specific member of the HSPA family, was thus presumed to be under the control of factors specific for spermatogenic cells. In order to determine the DNA sequences required for such expression pattern, and due to lack of suitable in vitro models, we constructed TG mice bearing a transgene composed of a CAT reporter fused to various fragments of the rat *HSPA2* gene promoter. This approach allowed to establish that a relatively short fragment of DNA (−367 to −203 nt in relation to ATG) located directly downstream of the T1 transcription start site (within the non-coding exon 1) is sufficient to drive testis-enriched and developmentally regulated expression of the transgene (Fig. 1). Within this region, we identified two short stretches which we termed box A (nt −346 to −325) and box B (nt −260 to −235) sharing the highest degree of nucleotide sequence homology between the mouse and rat genes. These boxes were shown to be of critical importance for transgene expression in the testis of TG mice (Fig. 1; Widłak et al. 1994; Widłak et al. 1995; Dix et al. 1996b; Scieglinska et al. 2001, 2004; Widlak et al. 2007a).

A search for potential *cis*-regulatory elements within the box A region led to the identification of a consensus sequence for binding of Sp1 transcription factor (GC-box) and a sequence highly similar to the octamer-binding transcription factor 1 binding site (Oct1-like). In turn, within the box B region, we found an estrogen-responsive element (ERE)-like sequence which differed by one nucleotide from the ERE and a H1t-like motif highly similar to the sequence involved in regulation of the *H1t* gene expression. Downstream of the box B region, just in front of the T2 transcription start site, a second GC-box is placed. The localization of these regulatory elements in the *HSPA2* promoter along with the results of the functional promoter analysis is summarized in Fig. 1.

Band shift analysis revealed that the testes of immature rats (9–10 days old), which do not express the *HSPA2* gene, contain some protein(s) that specifically interacts with the Oct1-like motif and the GC-boxes, respectively (Scieglińska et al. 2004; Widlak et al. 2007a). This observation indicates that some, so far unidentified protein(s), may act as repressor(s) of *HSPA2* gene expression in spermatogonia and early spermatocytes. Therefore, it is possible that dissociation of these repressors from the *HSPA2* promoter is a prerequisite for activation of the gene expression in pachytene spermatocytes. In regard to the TE-1 and ERE-like motifs of the box B, we found that these sequences are not involved in the regulation of *HSPA2* gene expression in testis (Scieglińska et al. 2004; Krawczyk et al. 1992). In summary, the available data allow us to hypothesize that in spermatocytes, the *HSPA2* gene could be under positive control of some general transcription factors (probably including their testis-specific variants). It is also probable that transcriptionally competent chromatin could be one of the main requirements for *HSPA2* gene expression. Such a supposition is partially supported by the observation that the *HSPA2* promoter is also active when present in an episomal form both in testicular and somatic cells (Widłak et al. 2003b; 2007a).

It is worth noting that the *cis* and *trans* elements involved in the *HSPA2* gene regulation are still poorly characterized. However, the identification of the promoter fragment required for testis-enriched expression enabled construction of TG mice in which expression of various proteins, such as CAT (Widłak et al. 1995; Scieglinska et al. 2001), EGFP (Widłak et al. 2003b; Rupik et al. 2006), heat shock transcription factor HSF1 (Widłak et al. 2003a; Vydra et al. 2006), heat shock protein HSPA1 (Widlak et al. 2007c), or Cre recombinase (Inselman et al. 2010), was effectively targeted into spermatocytes.

### Translational and posttranslational regulation of HSPA2 expression in rodent testis

Recent study has suggested that, in rodent testis, the expression of the *HSPA2* gene could be regulated at the translational and posttranslational levels by mechanisms that engage several different proteins. Hu antigen R (HuR), an RNA-binding protein, is known to influence the translation of multiple mRNAs by binding to their 3′ untranslated region and (among others) controls stability of long-lived mRNAs that are translated at late stages of spermiogenesis (Nguyen Chi et al. 2009). Aberrant expression of HuR protein (either deficiency or overexpression) in mice testis led to defective spermatogenesis manifested by massive death of spermatocytes (Chi et al. 2011). It has been found that HuR supports the optimal binding of the *HSPA2* mRNA to translating ribosomes in elongating spermatids and possibly also in pachytene spermatocytes (Chi et al. 2011).

HSPA2 expression in testis can be regulated at posttranslational level by proteins such as the following: the multifunctional Bat3 (HLA-B-associated transcript 3, also known as BAG6) chaperone belonging to BCL2-associated athanogene (BAG) domain protein family (Sasaki et al. 2008; Kawahara et al. 2013); HSPA binding protein HSPBP1, a nucleotide exchange factor of HSPA proteins and inhibitor of protein degradation mediated by the carboxy terminus of HSP70 interacting protein CHIP (Rogon et al. 2014); and eukaryotic translation initiation factor 4 gamma 3 (Eif4g3, Sun et al. 2010).

Bat3 and the co-chaperone HSPBP1 were shown to bind to HSPA2 and prevent its ubiquitination and proteasomal degradation in the testis (Sasaki et al. 2008; Rogon et al. 2014). Deficiency of Bat3, or HSPBP1, in knockout (KO) mice leads to male infertility caused by impaired spermatogenesis with morphological features similar to those observed in *HSPA2* null mice. It turned out that in the testis of these two knock-out mice strains, the level of HSPA2 protein is significantly reduced (Sasaki et al. 2008; Rogon et al. 2014). Bearing in mind that HSPA2 is expressed in certain somatic cells, it would be important to elucidate to what extent HSPA2 expression in non-testicular cells could be regulated by controlled degradation. Such a Bat3-dependent regulatory mechanism seems to exist because depletion of Bat3 was associated with decreased stability of HSPA2 in mouse embryonic fibroblasts (MEFs) and human teratocarcinoma cells (Sasaki et al. 2008). Also, the CHIP inhibitors HSPBP1 and BAG2 were shown to jointly control the proteasomal degradation of HSPA2 in MEF cells (Rogon et al. 2014).

Eif4g3, another protein which is supposed to modulate the level of HSPA2, is a multipurpose adapter connecting mRNA and ribosomes that acts as a scaffold protein for other factors involved in the formation of the initiation complex. Missense mutation in exon 36 of the mouse *Eif4g3* gene causes a failure of spermatocytes to exit meiotic prophase. Interestingly, the HSPA2 protein is absent from spermatocytes of *Eif4g3* mutant mice in spite of the presence of Bat3 and normal levels of the *HSPA2* mRNA (Sun et al. 2010). These authors hypothesized that Eif4g3 could be engaged in the translation of some unidentified regulator(s) of HSPA2 protein stability or be involved in initiation of HSPA2 translation.

### Regulation of the HSPA2 gene in human tissues

Mechanisms and factors that modulate the activity of the human *HSPA2* gene are even less understood than its rodent counterparts. Functional promoter analysis revealed the 392-nt-long DNA fragment localized directly upstream of the ATG codon to be the shortest promoter fragment sufficient to support transcription of the CAT reporter gene both in cancer cells expressing (A549) and non-expressing (HepG2) the endogenous *HSPA2* gene (Piglowski et al. 2007). These results indicate that the transcription factors necessary to support the *HSPA2* promoter activity at a basal level are contained in somatic cells irrespectively of the status of the endogenous gene activity, allowing speculation that epigenetic mechanisms could be of prime importance in the regulation of the *HSPA2* gene expression.

The accumulating evidence points to DNA methylation as one of the most important epigenetic mechanisms modulating the *HSPA2* expression level in human cells. *HSPA2* gene methylation was detected in numerous breast, cervical, bladder, and renal cancer cell lines (Ye et al. 2012; Pulverer et al. 2012; Costa et al. 2010; Sova et al. 2006). Since treatment of cells with 5-azacytidine (a DNA methylation inhibitor) restored *HSPA2* transcription to a high level (Ye et al. 2012; Sova et al. 2006), but hypermethylation induced the opposite effect (Costa et al. 2010), it seems probable that the methylation status of the *HSPA2* gene correlates with its transcription level. In genome-wide studies designed to unveil new bladder cancer-specific epigenetic markers, the *HSPA2* gene was found among the most frequently methylated genes both in bladder cancer cell lines and in primary bladder tumors, but not in normal urothelial tissue (Costa et al. 2010; Reinert et al. 2011). The frequent cancer-specific hypermethylation of the gene was also detected in endometrial and invasive cervical cancers (Fiegl et al. 2004; Widschwendter et al. 2004).

Although the above-mentioned observations strongly suggest that epigenetic modification could be important for regulation of *HSPA2* gene expression, there are some controversies regarding this issue. While hypermethylation-related repression of the *HSPA2* gene in primary bladder tumors has been reported (Costa et al. 2010; Reinert et al. 2011), others found the *HSPA2* gene to be highly expressed in bladder cancer cell lines and in primary urothelial tumors (Garg et al. 2010b)*.* Similar discrepancies have emerged in the case of HeLa (cervix) and MCF-7 (breast) cancer cell lines. Whereas high expression of the *HSPA2* gene in these cells was reported by several groups (Rohde et al. 2005; Daugaard et al. 2007; Garg et al. 2010a), in other works, the *HSPA2* gene was found to be hypermethylated and expressed at insignificant levels (Sova et al. 2006; Pulverer et al. 2012; Ye Ch et al. 2012). The reasons for these inconsistencies are unknown.

### The HSPA2 gene expression in response to heat shock and hypoxia

In somatic cells exposed to heat shock, a subset of HSP genes is activated by binding of the HSF1 transcription factor to the regulatory heat shock elements (HSEs) present in promoters of stress-inducible genes. Rodent orthologs of the *HSPA2* gene contain HSE-like sequence of low similarity to the canonical HSE and does not bind constitutively active mutant of HSF1 (Widlak et al. 2007b). The *HSPA2* gene expression is not induced by heat shock in rodent spermatogenic and somatic cells (Krawczyk et al. 1988; Zakeri et al. 1988; Zakeri et al. 1990). Also, the human *HSPA2* gene is not induced by heat shock (Scieglińska et al. 2008; Hageman et al. 2011), and no binding of HSF1 to the *HSPA2* promoter was detected in heat-shocked human cells (Trinklein et al. 2004; Hageman et al. 2011; Vihervaara et al. 2013). Noteworthy, up-regulation of *HSPA2* gene expression*,* likely mediated rather by HSF2 and not HSF1, was observed in K562 erythroleukemia cells upon treatment with hemin, a differentiation-inducing metabolite (Trinklein et al. 2004).

Increasing the temperature of rat testes by placing them in the abdominal cavity (experimental cryptorchidism) results in rapid disappearance of the HSPA2 mRNA and degeneration of seminiferous epithelium (Krawczyk et al. 1987a; Widlak et al. 2007b). Similarly, a marked reduction of the *HSPA2* expression level occurred in parallel with rapid degeneration of seminiferous epithelium due to massive apoptosis of spermatocytes when the constitutively active mutant of HSF1 was overexpressed in testes of transgenic mice (Nakai et al. 2000; Widłak et al. 2003a; Vydra et al. 2006). At present, the mechanism by which HSF1 affects *HSPA2* gene expression is unknown. HSF1 could possibly suppress some genes which encode so far unidentified factors crucial for HSPA2 expression in testis (Widlak et al. 2007b). The detrimental influence of HSF1 on the seminiferous epithelium demonstrates that the response of somatic and spermatogenic cells to heat shock is clearly different. This dissimilarity also manifests itself in the observation that the forced overexpression of HSPA1 in spermatocytes of TG mice shows no protective effect against apoptosis induced either by experimental cryptorchidism or by overexpression of active HSF1 (Widlak et al. 2007c).

The study of cancer cells pointed to the possible activation of the HSPA2 expression by hypoxia, a condition frequently associated with tumor propagation. In fact, hypoxia increased the HSPA2 expression in human hepatoma (HepG2), breast (MCF-7), and cervical (HeLa) cancer cells. Functional promoter studies and chromatin immunoprecipitation assays confirmed that the activation of the *HSPA2* gene was mediated by interaction of hypoxia-inducible factor (HIF)-1 transcription factor with the hypoxia-responsive element (HRE) localized at 446-bp upstream of the transcription start site (Huang et al. 2009). Our recent study revealed that *HSPA2* gene expression can also be modulated by HIF-1 and hypoxia in keratinocytes, but in contrast to HepG2 cells, both primary keratinocytes and the immortal human keratinocyte line HaCaT respond to hypoxia by a significant decrease in the *HSPA2* gene expression level (our unpublished results). This observation seems relevant in the context of a crucial role of HIF-1 in the maintenance of skin homeostasis (Cho et al. 2008; Rezvani et al. 2011). Physiological oxygen pressure in the epidermis ranges between 0.2 and 8 %, and thus, moderate hypoxia is a physiological condition during growth and differentiation of keratinocytes (Rezvani et al. 2011; Evans et al. 2006). Consequently, one can assume that HIF-1-dependent modulation of *HSPA2* gene expression in keratinocytes might be related to normal processes of development/differentiation of skin epithelium.

## Function of the HSPA2 protein in spermatogenesis

Phenotype analysis of *HSPA2* KO mice led to the conclusion that the *HSPA2* gene is crucial for male, but not female, fertility (Dix et al. 1996a). HSPA2 deficiency led to severe degeneration of seminiferous epithelium manifested by massive apoptosis of pachytene spermatocytes and lack of postmeiotic cells (Dix et al. 1996a, 1997). Profiling the expression of molecular markers specific for defined stages of spermatogenesis established that *HSPA2* depletion severely impaired the late stages of spermatocyte development (Dix et al. 1997).

The HSPA2 protein was found to localize along the structures formed by synaptonemal complexes in pachytene spermatocytes of wild-type mice (Allen et al. 1996). Microscopic observation of the testis of *HSPA2* null mice suggested that HSPA2 is required for desynapsis of synaptonemal complexes (Dix et al. 1997). In *HSPA2* KO males, spermatogenesis arrested at G2/M and did not progress to the M stage of meiosis. Subsequent analysis revealed that HSPA2 is indispensably required for the formation of the cyclin-dependent kinase 1 (CDK1)/cyclin B1 complex in spermatocytes and confirmed its essential role for progression of meiosis. HSPA2 was found to interact with CDK1, but not with cyclin B, and only when the kinase was not complexed with cyclin B1 (Zhu et al. 1997).

The HSPA2-dependent regulation of CDK1/cyclin B1 activity seems to be modulated by at least two proteins: H1t, a testis-specific variant of linker histone H1, and tNASP, a testis/embryo form of nuclear autoantigenic sperm protein (Alekseev et al. 2009). The latter belongs to a family of specific histone chaperones which facilitate the incorporation of linker histones into chromatin and enable the progression of the cell cycle through the G1/S border (Finn et al. 2012). Intriguing data indicate the occurrence of interaction between tNASP, H1t, HSPA2, and CDK1 in mouse spermatocytes. H1t binds to the tNASP-HSPA2 complex. This interaction enhances weak intrinsic HSPA2 ATPase activity, increases the ability of HSPA2 to tether CDK1, and concomitantly prevents the binding of cyclin B1 to HSPA2/CDK1 complexes. Altogether, the coordinated action of tNASP and histone H1t modulates the ability of HSPA2 to form an active CDK1/cyclin B1 complex and regulates the progression of meiosis (Alekseev et al. 2009).

Experimental data suggest that during mouse spermiogenesis, HSPA2 also participates in the chromatin condensation. At the beginning of this multistage process, both core and linker histones are replaced by their testis-specific variants. Concomitant, massive hyperacetylation of chromatin is believed to destabilize its nucleosomal structure and to facilitate the replacement of testis-specific histones by transition proteins (TPs) and finally by other basic DNA-packaging proteins (Gaucher et al. 2010)*.* Proteomic analysis and microscopic visualization revealed that in elongating spermatids, HSPA2 undergoes redistribution from a dispersed intranuclear location to subacrosomal domains, and these changes correlated with the cessation of histone acetylation and histone removal (Govin et al. 2006). Detection of complexes containing TP1, TP2, and HSPA2 led to the conclusion that HSPA2 performs a role of a specific chaperone for DNA-packaging TPs, being involved in the assembly of new spermatid-specific nuclear structures (Govin et al. 2006).

The HSPA2-dependent replacement of histones by TP is supposed to be modulated by poly(ADP-ribose) polymerases PARP-1 and PARP-2. These enzymes belong to the large PARP family, members of which contribute to the epigenetic regulation of various physiological processes including chromatin reorganization during spermiogenesis (Dantzer et al. 2006; Quenet et al. 2009; Meyer-Ficca et al. 2009). Mass spectrometry analysis revealed that in mouse spermatids, PARP-2 interacts with HSPA2 in a TP2-dependent manner and PARP-1 modifies HSPA2 structure by ADP-ribosylation (Quenet et al. 2009). These results suggest that HSPA2, together with TP2, PARP-1, and PARP-2, could form spermatid-specific complex(es), presumably of crucial importance for chromatin remodeling. Likely, coordinated processes of PARP-1-dependent poly(ADP-ribosyl)ation of HSPA2, PARP-2-dependent poly(ADP-ribosyl)ation of TP, and chaperoning activity of HSPA2 exerted on TP2 and TP1 might contribute to chromatin reorganization and formation of spermatozoa (Quenet et al. 2009).

There is some indirect evidence implying that the spermatid-specific chromatin remodeling can be affected by phosphorylation of HSPA2. According to the PhosphoSitePlus (http://phosphosite.org) database, HSPA2 can be phosphorylated at some serine and threonine residues in vivo. Moreover, HSPA2 was found to be among potential targets of serine/threonine phosphoprotein phosphatase 1 gamma 2 (PPP1CC2; Henderson et al. 2011). Expression of PPP1CC2, one of the two alternatively spliced isoforms encoded by the *Ppp1cc* gene, is restricted to spermatogenic cells. Disruption of the *Ppp1cc* gene results in aberrant spermiogenesis manifested by abnormal chromatin packing (histone retention), increased germ cell apoptosis, and, consequently, deficiency of condensing and elongating spermatids (Varmuza et al. 1999; Forgione et al. 2010). Taking into account that PPP1CC2 can directly interact with HSPA2 and PPP1CC2 depletion leads to the accumulation of hyperphosphorylated HSPA2 in testis, it was proposed that the PPP1CC2-dependent modulation of HSPA2 structure/activity could contribute to chromatin remodeling during spermiogenesis in mouse (Henderson et al. 2011).

The above data suggest that in mice (and possibly in other rodents), posttranslational modifications of HSPA2 could be crucial for the process of chromatin remodeling, presumably switching the ability of HSPA2 to interact with specific client proteins during different stages of spermiogenetic maturation. Such an intriguing possibility should be however experimentally verified.

A number of biochemical and clinical observations show that also in humans, expression of the *HSPA2* gene is required for male fertility. However, the results of studies published so far suggest that the expression pattern and functions of HSPA2 in rodent and human spermatogenesis may not fully overlap. In the human testis, HSPA2 is expressed at a significantly higher level in elongating spermatids and spermatozoa than in spermatocytes (Huszar et al. 2000; Motiei et al. 2013; Redgrove et al. 2012). So far, neither the role of human HSPA2 in the dissociation of the synaptonemal complex nor its chaperoning activity exerted on the components of cyclin-CDK complexes has been documented. Instead, adequate evidence points to a crucial role of HSPA2 in spermiogenetic maturation and sperm ability to fertilize an oocyte.

Early studies linked the reduced levels of HSPA2 (initially described as an unusual variant of creatinine kinase M) with aberrant spermiogenetic maturation and production of non-functional sperm characterized by retention of cytoplasm, aberrant plasma membrane remodeling, and lack of *zona pellucida* (ZP) binding sites (Huszar et al. 2000; Huszar et al. 1997; Huszar et al. 1998). Low level of HSPA2 was also correlated with increased aneuploidy frequencies in immature sperm. The type of aneuploidy (disomies) allowed to speculate that low HSPA2 expression might be related to impaired meiotic division of human spermatocytes (Kovanci et al. 2001).

In subsequent studies, reduced expression of the *HSPA2* gene was found in the testes or in spermatozoa of men with abnormal spermatogenesis (Feng et al. 2001; Cedenho et al. 2006; Lima et al. 2006; Terribas et al. 2010). In infertile men, also a low number of HSPA2-positive sperm were detected (Motiei et al. 2013). Recently, an association between impaired fertilizing ability of sperm from patients diagnosed with idiopathic male infertility and a total lack of HSPA2 in their sperm has been reported (Redgrove et al. 2012).

There are discrepancies between results on HSPA2 localization in sperm. Motiei et al. (2013) have shown that sperm maturation is associated with rearrangements of HSPA2 localization from intracellular in non-capacitated sperm, to superficial in capacitated spermatozoa. Other group has shown that HSPA2 localizes solely in intracellular sperm compartments regardless of the capacitation status (Redgrove et al. 2012; 2013)*.* However, despite these controversies, it seems evident that HSPA2 is important for sperm-egg recognition. Early studies demonstrated that a reduced level of HSPA2 in spermatozoa is manifested by decreased ability of sperm to interact with oocyte and to fertilize it (Huszar et al. 1994). Diminished sperm HSPA2 levels were shown to predict in vitro fertilization failure (Ergur et al. 2002). According to recent studies performed by Redgrove et al. (2012; 2013), HSPA2 is a component of a multimeric sperm-egg recognition complex which contains sperm adhesion molecule 1 (SPAM1) and arylsulfatase A (ARSA) proteins, both previously implicated in sperm-oocyte interactions. The authors proposed that SPAM1 is positioned on the membrane of non-capacitated sperm to mediate binding and dispersion of the hyaluronic-rich matrix of the cumulus mass. As sperms penetrate the cumulus mass and finish capacitation, the complex is reoriented to express ARSA, one of the putative receptors for the ZP. In this model, HSPA2 is localized intracellularly, and its activity could provide a driving force for SPAM1 and ARSA movements. Investigation of the dynamic structure of the HSPA2/ARSA/SPAM1 complex also pointed to potential capacitation-dependent phosphorylation of HSPA2 mediated via activity of protein kinase A (Redgrove et al. 2013). This further implies that posttranslational modifications of HSPA2 could have a crucial impact on its activity. Although the results cited above point to an essential role for HSPA2 in the sperm-egg recognition process, a comprehensive understanding of its function in the fertilization process still requires further studies.

## Function of HSPA2 in normal somatic and cancer cells

At present, the function of HSPA2 in human somatic cells is poorly characterized and can be only inferred from a few studies. Two recent studies have demonstrated that HSPA2 exhibits chaperoning and cytoprotective activity. Hageman et al. (2011) have shown that overexpression of HSPA2 in human embryonic kidney HEK-293 cells enhanced refolding of heat-denatured luciferase and suppressed formation of luciferase aggregates in heat-shocked cells. We have reported that overexpression of HSPA2 in Chinese hamster lung fibroblasts (V79 cells) increased the resistance of cells to apoptosis induced by heat shock or proteasome inhibitors (Filipczak et al. 2012). Interestingly, HSPA2 did not protect cells against toxic effects caused by the microtubule-affecting drugs vinblastine and paclitaxel or by the DNA-intercalating topoisomerase II inhibitor doxorubicin. Therefore, one can speculate that HSPA2 has selective cytoprotective activity. HSPA2 could also influence the function/structure (but not stability) of lysosomal/endosomal membranes. Such a supposition emerged from the observation that extracellularly supplied recombinant HSPA2 co-localized with markers specific for late endosomes and lysosomes (Kirkegaard et al. 2010).

Although expression of the *HSPA2* gene is not induced by heat shock (Scieglińska et al. 2008; Hageman et al. 2011), a sudden increase in temperature causes rapid translocation of HSPA2 from the cytoplasm into the nuclei and nucleoli as well as into the area of centrosomes (Scieglińska et al. 2008). Such a pattern of HSPA2 translocation is similar to that observed for the heat-inducible HSPA1 and HSPA6 proteins (Pelham 1984; Hut et al. 2005; Khalouei et al. 2014). This suggests that HSPA2 may participate in the protection of nucleolus and/or centrosome integrity under conditions of thermal (and possibly other) stress(es) (Scieglińska et al. 2008), as suggested earlier for HSPA1 (Pelham 1984; Hut et al. 2005).

The majority of the knowledge on the HSPA2 function in cancer cells has been inferred from studies in which the expression of the *HSPA2* gene was silenced by RNA interference (RNAi) technology (Rohde et al. 2005; Daugaard et al. 2007; Garg et al. 2010a; 2010b). The earliest studies revealed that even single transfection of HeLa or MCF-7 cells with siRNA targeted against *HSPA2* mRNA induced a senescence-like phenotype and G1 cell cycle arrest followed by apoptosis-like cell death (Rohde et al. 2005; Daugaard et al. 2007). Because HSPA2 depletion had no effect on proliferation of non-cancerous immortalized epithelial cells derived from breast or prostate epithelia, it has been concluded that HSPA2 is specifically required for growth of cancer cells (Rohde et al. 2005). The importance of HSPA2 for cancer cell proliferation has been underlined by observations showing reduced growth of human urothelial and cervical cancers cells in response to HSPA2 depletion (Garg et al. 2010a, b). Moreover, Garg et al. (2010a, b) reported that *HSPA2* knockdown decreased migration of cancer cells.

The results described above are in contrast with the effects of HSPA2 depletion in non-small cell lung cancer (NSCLC) cell lines that were obtained by our group. By stably infecting NSCLC cells using viral vectors (lentiviruses or retroviruses) encoding small hairpin RNAs (shRNAs), we effectively reduced *HSPA2* gene expression by up to 90 % of the controls (non-infected or infected with scrambled shRNA). However, no effects on cell viability have been detected in HSPA2-depleted cells (Scieglinska et al., unpublished). Because each study cited above exploited a different cancer cell line and different gene silencing strategies, it is evident that more systematic studies are needed in order to understand the cause of the variable effects of HSPA2 deficit on growth of cancer cells.

One of the most intriguing issues concerning the potential influence of HSPA2 on the phenotype of tumor cells emerges from the comparison of the HSPA2 expression pattern in primary tumors and in the corresponding normal tissues (Rohde et al. 2005; Garg et al. 2010b; Scieglinska et al. 2014). In the case of certain tumors, as exemplified by breast and bladder cancers, HSPA2 can be detected in tumor but not in the corresponding normal tissue (Garg et al. 2010b; Scieglinska et al. 2011, 2014)*.* It therefore seems evident that in these cases, the expression of the *HSPA2* gene is initiated during tumor development. On the other hand, HSPA2 expression can be detected at comparable levels both in tumor and corresponding normal tissue as exemplified by lung squamous cell cancer (SCC) tumors and normal bronchial epithelium (Scieglinska et al. 2011, 2014)*.* Thus, one can presume that oncogenic transformation from normal bronchial epithelium to SCC lung cancer is accompanied by continued expression of the *HSPA2* gene (Scieglinska et al. 2011). Finally, all of the observations above allow to propose that HSPA2 could play different roles in various types of cancer, depending on whether this protein was expressed in cancer precursors or its expression was acquired during oncogenic transformation.

Another interesting aspect of the HSPA2 expression in tumors concerns possible functional differences between this protein and HSPA1, another member of the HSPA family. HSPA1 is a major stress-inducible protein which is frequently overexpressed in various tumors. A selective silencing of either HSPA2 or HSPA1 in HeLa cells resulted in distinct phenotypes. While HSPA2-depleted cells acquired a senescent-like phenotype and were arrested in G1 of the cell cycle, silencing of the *HSPA1* gene resulted in G2/M arrest and immediate entry of cells into apoptosis. Also, a global transcriptome analysis of HeLa cells revealed strikingly distinct changes in gene expression between HSPA2- and HSPA1-depleted cancer cells (Rohde et al. 2005; Daugaard et al. 2007). These observations suggest that HSPA2 and HSPA1 exhibit substantial functional diversity and, possibly, they can be a part of distinct functional networks and/or they can interact with diverse arrays of client proteins.

The role of HSPA2 in rodent somatic cells is entirely unknown. Expression of the *HSPA2* gene was observed in major developmental events occurring during mouse embryogenesis (Murashov and Wolgemuth 1996a; Rupik et al. 2006). Although these results would indicate an involvement of HSPA2 in multiple processes during mouse embryogenesis, the deletion of the *HSPA2* gene from mouse genome was not associated with reduced litter size, developmental abnormalities in newborn mice, or increased perinatal mortality as shown earlier by Dix et al. (1996a), indicating that HSPA2 is dispensable for mouse embryo development. At present, the cause of this apparent discrepancy is hard to explain. Since the HSPA2 protein has been found in embryonic mouse tissues, one can assume that the HSPA2 deficiency during development of *HSPA2* KO mice could be substituted by other structurally and functionally similar protein(s).

## Concluding remarks

The features of the *HSPA2* gene, although it has been studied for more than 25 years, are still poorly characterized. Presumably, the reason is the long-lasting conviction that the *HSPA2* gene is selectively involved in sperm cell differentiation, maturation, and activity. The recent studies have demonstrated, however, that the *HSPA2* gene can be expressed at a relatively high level in selected populations of somatic cells. These findings allow us to assume that HSPA2 may have some, yet unidentified, functions (possibly including behavioral or cognitive) that cannot be directly inferred from observations of *HSPA2* KO mice. Recent studies clearly show that the level of HSPA expression can be regulated by epigenetic (promoter methylation) and posttranscriptional mechanisms.

The intriguing issue concerns the role of the *HSPA*2 gene in human tumor cells. Although the expression of the *HSPA2* gene in tumors is unquestionable, the influence of HSPA2 on the phenotype of cancer cells has not been unequivocally established. One of the most important issues is to determine to what extent HSPA2 influences survival and proliferation of cells of various cancer histotypes. So far, in numerous studies aimed at correlating the constitutive expression of HSPA (e.g., HSPA1 and HSPA8) with clinical features, the possible co-expression of the *HSPA2* gene was, for obvious reasons, not taken into consideration. Bearing in mind that these proteins share several properties, the question concerning the similarity of their influence on cancer cell proliferation, resistance to cell death signaling, immunogenic properties, etc. is important. A clear answer to this question can be critical for verification of the potential usefulness of HSPA2, as well as other HSPA proteins, as clinical biomarkers. So far, there are no data concerning HSPA2 expression in tumors and tumor cells of rats and mice. Thus, it is unknown whether rodent models (except xenografts) would be suitable to study the role of HSPA2 in cancerogenesis. We hope that the data summarized in this review show clearly that HSPA2 represents an intriguing multifunctional member of the HSPA family.
